# Conscious Action/Zombie Action

**DOI:** 10.1111/nous.12086

**Published:** 2015-01-09

**Authors:** Joshua Shepherd

**Affiliations:** ^1^ University of Oxford

## Abstract

I argue that the neural realizers of experiences of trying (that is, experiences of directing effort towards the satisfaction of an intention) are not distinct from the neural realizers of actual trying (that is, actual effort directed towards the satisfaction of an intention). I then ask how experiences of trying might relate to the perceptual experiences one has while acting. First, I assess recent zombie action arguments regarding conscious visual experience, and I argue that contrary to what some have claimed, conscious visual experience plays a causal role for action control in some circumstances. Second, I propose a multimodal account of the experience of acting. According to this account, the experience of acting is (at the very least) a temporally extended, co‐conscious collection of agentive and perceptual experiences, functionally integrated and structured both by multimodal perceptual processing as well as by what an agent is, at the time, trying to do.

## 1. Introduction

At some point many of us have performed the following exercise. Concentrating on what it is like to do it, we decide to move a body part and move it, slowly and carefully. Perhaps we look at our hand, lifting it into the air for a moment, before deliberately wiggling each finger. Doing so, it certainly *appears*—in some very vague way—like consciousness is playing a major causal role.

Does consciousness really play a major causal role in action control? This question needs refinement. Often experiences of action—or at least experiences closely associated with the performance of an action—come in more than one modality. Such experiences might be visual, proprioceptive, or auditory, and might involve cognitive or agentive elements as well. In section two I argue that at least one experience‐type—the experience of trying—does play a major causal role in action control. This is because the neural activities that subserve experiences of trying are not distinct from the neural activities that subserve actual tryings.

This raises a question regarding the causal role of various perceptual experience‐types, and how they might relate to experiences of trying. A number of philosophers and cognitive scientists have argued that the role of conscious vision for action control is much less important than commonsense indicates. According to these writers, though conscious vision might seem important for action control, it is “zombie systems”—systems that run outside the scope of conscious awareness—that do much of the work. In section three I assess the scope and philosophical importance of arguments for zombie action. I clarify the challenge from zombie action, and I discuss three ways the phenomenal character of visual experience might be implicated in it. In section four I argue that strong versions of zombie action are false. Conscious visual experience does sometimes play an important causal role for action control.

In section five I propose and defend an account of the experience of acting that begins to explain how experiences of trying relate to perceptual experiences in action. According to the account I defend, the experience of acting is (at the very least) a temporally extended, co‐conscious collection of agentive and perceptual experiences, functionally integrated and structured both by multimodal perceptual processing as well as by what an agent is, at the time, trying to do.

## 2. The Experience of Trying

Consider lifting a heavy weight with one's arm. Doing so, one will often experience tension in the elbow, strain or effort in the muscles, heaviness or pull on the wrist, and so on. In addition, there is an aspect of this experience that is not to be identified with any of these haptic elements, or with any conjunction of them. When lifting the heavy weight, one has an experience of trying to do so. Put generally, the experience of trying is an experience as of directing effort (however minimal) towards the satisfaction of an intention (this is not to say that possessing a concept of intention or of an intention's satisfaction is necessary for the capacity to have such experiences). In the example at hand, it is a phenomenal character as of directing effort towards the movements of the arm.

I am not sure how much credence to give to my own phenomenology here, but I have experiences as of directing effort towards the satisfaction of a wide range of intentions. These experiences include directing effort towards (a) fixing attention on a miniscule feature of conscious perception (whether visual, auditory, haptic, or whatever), (b) clenching and unclenching my fist, (c) squatting down to the ground and then standing, and many more.^1^ To be clear, these experiences of directing effort do not accompany every intentional action I perform, nor do they accompany every moment of the performance of many intentional actions. Nor are they simply experiences of initiating action. Although the experience of trying sometimes seems to coincide with the initiation of action, it can accompany stages of action execution beyond initiation, and this experience often extends temporally throughout significant periods of action execution. Further, these experiences of trying do not seem (to me) to be tied to any particular amount of effort. What is essential to the experience, in my view, is the *direction* of effort.

One way to better understand the directive nature of experiences of trying is by contrast with a type of experience recently discussed by Susanna Siegel (2014). According to Siegel, some perceptual experiences might be experienced as *mandates*. As Siegel explicates the possibility, experienced mandates are a type of action‐affordance—that is, in this context, an action‐possibility present in an agent's environment that the agent consciously perceives as an action‐possibility. For Siegel, experienced mandates are experienced affordances which include “a high degree of felt solicitation” (2014, 55) to perform some relevant action. For example:

[S]uppose in the midst of an important conversation the tuft of hair keeps falling over your interlocutor's eye, obstructing proper communication by interfering with eye contact. You might well experience the hair as an obstacle that should be moved away to allow for fuller eye contact. (54)

Experienced mandates are *perceptual* experiences of action‐affordances that include an experience of “felt solicitation” towards action. When we experience an itch, for example, at least some of the time we experience the itch as soliciting an action (a scratch). In such a case it is a sensory experience as of some bodily location itching that compels the action. The directive character of experiences of trying, by contrast, does not emanate from any bodily location. It is not incorrect to call it an experiential mandate. But in this case the mandate seems to emanate from the agent. When I have an experience of trying to raise my arm, I have an experience as of mandating that my arm rise. It is this fundamentally directive character that marks the experience out as an experience of trying.

Consider two episodes of walking. In the first, one has an experience of trying to walk (in addition to broadly haptic experiences related to the legs and body moving). In the second, one walks without having an experience of trying to do so. I frequently have both kinds of experiences: although I frequently experience myself directing effort towards the steps I make, when on a long walk it is possible to direct my attention and my efforts elsewhere while still walking. At such points it seems to me like I am directing no effort towards the steps I am making. It seems as though my legs move more or less of their own accord, and it seems this way precisely because the experience of trying to move my legs is absent (cf. Peacocke 2007).

A further point regarding the experience of trying deserves mention. It is well known that the awareness we have of our bodies in motion is relatively rough‐grained. For example, while acting one often makes fine‐grained motor adjustments, but one is rarely aware of these adjustments. In my view this is consistent with the content of a typical experience of trying. The experience of directing effort towards, e.g., movements of the arm, does not entail that one experiences the direction of effort towards every minute movement the arm makes. Indeed, it is often the case that many of the movements made are only roughly in accordance with the effort experienced as directed.

It is worth noting here that this does not entail the causal irrelevance of experiences of trying. The best current models of action control emphasize the importance of hierarchical feedback loops (see, e.g., Grafton and Hamilton 2007, Shepherd forthcoming). According to these models, the kind of low‐level processes that subserve fine‐grained motor adjustments are driven by goals that are distinct from (though embedded within) higher‐level goals and plans, operate on different informational inputs than do higher‐level processes, respond to different kinds of feedback than do higher‐level processes, and have limited communication with higher‐level processes (for a clear exposition of these ideas and evidence in favor of them, see Logan and Crump 2011). It remains possible that the neural activity responsible for experiences of trying exists at a higher level in the action control hierarchy than the neural activities responsible for fine‐grained adjustments and low‐level motor programming.

That the experience of trying exists I take to be manifest. No doubt some philosophers will disagree^2^: below I offer evidence (beyond my own phenomenology) in favor of the existence of an experience of trying. However, arguing in favor of the existence of this experience‐type is not my only aim here. I also want to know whether experiences of trying play the causal role that they seem to play—that of directing effort towards the satisfaction of an intention.

In order to answer this, we need to reflect on the relationship between experiences of trying and what we can call actual tryings. Experiences of trying are, as I have said, experiences as of directing effort towards the satisfaction of an intention. Actual tryings are (not necessarily conscious) directions of effort (however minimal) towards the satisfaction of an intention.^3^ How are experiences of trying related to actual tryings?

A quick caveat: this question might invoke worries about mental causation for some readers. But the nature of mental causation is not one taken up by any of my interlocutors in what follows. I share with my interlocutors the assumption that conscious experiences have some causal impact. The questions I am interested in concern the relevance of that impact to action control. For present purposes, then, I speak in terms of the neural realizers of the experience of trying.

This caveat noted, we can draw a distinction between two kinds of views about the relation between experiences of trying and actual tryings. First, one might maintain that experiences of trying are distinct from actual tryings. More precisely, one might claim that the neural activities (i.e., the neural mechanisms, states or processes) that realize experiences of trying are distinct from the neural activities that realize actual tryings.^4^ (Perhaps experiences of trying merely signal or report the existence of trying—much in the way that visual experiences of the sky signal or report the existence of the sky.) Call this the non‐constitutive view.

Second, one might maintain that experiences of trying are not distinct from actual tryings. More precisely, one might claim that the neural activities that realize experiences of trying are not distinct from the neural activities that realize tryings. According to this claim experiences of trying, when they occur, are (at least partially) constitutive of tryings. Call this the constitutive view.

How do we decide between a constitutive and a non‐constitutive view? There are two prominent views of agentive experience that might seem to support a non‐constitutive view. I turn, then, to these views.

The first is Daniel Wegner's much‐discussed view that so‐called “conscious will” is an illusion. According to Wegner (2002), the illusion arises as a result of an inference agents make: in paradigmatic instances of the illusion, agents have a conscious thought that they are about to act, then perceive their bodies doing the things they thought their bodies would do, and infer that the movements were the result of conscious will. But, according to Wegner, conscious will plays no causal role in the bodily movements. According to Wegner, “unconscious and inscrutable mechanisms create both conscious thought about action and the action, and also produce the sense of will we experience by perceiving the thought as cause of the action” (2002, 98).

This is a kind of non‐constitutive view about the relation between what Wegner calls conscious will and the mechanisms that produce action. Does this view apply to experiences of trying? It is difficult to tell. As Bayne (2006) has argued, it is not transparent what Wegner means by the term “conscious will.” In places he calls it a feeling or an emotion, and in the above quote he asserts that some *sense* of will is produced by a perception that a thought is the cause of an action. This is on its own quite hard to parse, and at any rate it is difficult to reconcile with the fact that Wegner's account of the generation of conscious will makes conscious will seem more like a judgment – the result of an inference an agent makes. However, the experience of trying is not well described as a feeling, emotion, or judgment. Indeed, it looks like Wegner developed his model to explain something else entirely – something that might be called “a feeling of having been in control of an action.” So it is not clear that Wegner intends his arguments to apply to experiences of trying.

Even so, one might argue that the evidence Wegner advances in favor of his view in fact supports a non‐constitutive view about experiences of trying. Such an argument faces an uphill battle: in response to Wegner, a number of critics have argued that the evidence Wegner adduced for his view does not in fact support the view (see Nahmias 2002, Bayne 2006, Shepherd 2013, Walter 2014). There is not space to review the evidence, or the criticisms, here. Instead, in what follows I go on the offensive. I will argue that Wegner's view cannot accommodate work more directly relevant to experiences of trying, and indeed, that such work supports a constitutive view. Before I do so, however, I need to introduce the second non‐constitutive view of agentive experience. I need to do so because, as we will see, the empirical work I go on to discuss undermines this view as well as Wegner's.

The second non‐constitutive view involves appeal to a popular control‐theoretic model of overt (that is, bodily) action control: what many call the *comparator model*. According to the comparator model, an intention produces overt action by interacting with a tangled series of modeling mechanisms that take the intention's relatively abstract specification of a goal‐state and transform it into various fine‐grained, functionally specific commands and predictions. An *inverse model* (or ‘controller’) takes the goal state as input and outputs a motor command designed to drive the agent towards the goal‐state. A *forward model* receives a copy of the motor command as input and outputs a prediction concerning its likely sensory consequences. Throughout action production, the inverse model receives updates from various *comparator* mechanisms. On standard expositions of the model (e.g., Synofzik et al. 2008), three types of comparator mechanism are posited. One compares the goal‐state with feedback from the environment, and informs the inverse model of any errors; a second compares the goal‐state with the forward model's predictions, and informs the inverse model of any errors; a third compares the forward model's prediction with feedback from the environment, and informs the forward model (so as to develop a more accurate forward model).

Regarding the exercise of fine‐grained motor control, the comparator model is an explanatory success. Whether it has the resources to explain agentive experience is more controversial (see Mylopoulos 2012). On a comparator account of agentive experience, the comparator that sends information concerning predictive accuracy back to the forward model, as well as the comparator that sends information to the inverse model concerning the relation between predicted and desired states get added functions. When predicted and desired (or, at slower time scales, predicted and actual) states *match*, the given comparator ‘codes’ the activity as self‐generated. This code is then sent to a system hypothesized to use it in generating the sense of agency (Blakemore and Frith 2003). Proponents of the comparator account recognize that this is not a complete explanation of agentive experience, but they maintain that this matching process “lies at the heart of the phenomenon” (Bayne 2011, 357).

As with Wegner's view, this view seems clearly to support a non‐constitutive view. According to the comparator account, the neural activities that realize agentive experience are at one remove (at least) from the neural activities actually involved in action generation and control: agentive experience is constructed by a system that takes as input signals that report on the general workings of various action control mechanisms. More specifically, when predicted sensory feedback matches *actual* sensory feedback, a ‘comparator mechanism’ is posited to output a match signal that is used by a further mechanism to construct the sense of agency. An anonymous referee notes that a natural way to construe agentive experience on this model is in terms of judgments the agent makes about her action after the action has begun—judgments either constituted by or based upon the outputs of a mechanism that is functionally unimportant for the generation and direction of bodily movements. If this is right, then we might doubt that experiences of trying play the causal roles they seem to play.

Again, though, as with Wegner's view, it is worth noting that this account was not designed to explain experiences of trying. Rather, the target of this account is the “sense of agency”—a term of art that refers to agentive experience more broadly (as Sarah‐Jayne Blakemore and Chris Frith have it, the sense of agency is “the feeling that we cause movements and their consequences” (2003, 221)). Can a comparator account be extended to experiences of trying?

Two very interesting self‐paralysis studies indicate that the answer is no. In the first, Simon Gandevia and colleagues paralyzed themselves with atracurium, a neuromuscular block that left them fully conscious (Gandevia et al. 1993). While paralyzed participants attempted to move various limbs. They were asked “to concentrate on sensations associated with the contracting muscles while blood pressure and heart rate were monitored” (90). Gandevia et al. report:

All reported strong sensations of effort accompanying attempted movement of the limb, as if trying to move an object of immense weight. Subjective difficulty in sustaining a steady level of effort for more than a few seconds was experienced, partly because there was no visual or auditory feedback that the effort was appropriate, and because all subjects experienced unexpected illusions of movement. As examples, attempted flexion of the fingers produced a feeling of slight but distinct extension which subsided in spite of continued effort, and attempted dorsiflexion of the ankle led to the sensation of slow plantar flexion. Further increases in effort repeatedly caused the same illusory movements. (97)

Participants had experiences of trying to move a finger or ankle in a certain direction. And participants had experiences of the relevant finger or ankle moving in the other direction. This indicates that the experience of trying is both causally linked with and distinct from the experience of the body moving. Interestingly, one participant reported that the illusory experience of the body part moving would fade with time, but that an increase in the amount of effort deployed to move the finger or ankle would again make the illusion vivid.

Why would the neural activity associated with trying to move the finger cause an experience of the finger moving in the opposite direction? Gandevia et al. hypothesized that the paralyzing agent atracurium failed to fully block feedback from some muscle spindle afferents. In a follow‐up, they fully paralyzed these afferents via ischaemia (the cutoff of blood). After ischaemia, experiences of trying remained, but illusions of movement did not.

In the second self‐paralysis study, Robert Lansing and Robert Banzett (1993) paralyzed^5^ four healthy adult volunteers with doses of the neuromuscular blocking agent vecuronium. Lansing and Banzett asked participants (a) to make maximum voluntary efforts of inhaling against a closed airway at their end‐expiratory volume (i.e., after an exhaling), (b) to make a maximum contraction of thumb flexors, and (c) periodically to make other efforts to move at their own discretion. Participants were then placed through a 30–45 minute structured interview, during which they reported upon aspects of their experience, including “what sensations they experienced when they attempted maximum inspirations, maximum thumb flexions . . . or other movements” as well as “the location of any reported sensations of force or effort” (310).

Lansing and Banzett report that though “All subjects were sure that they had attempted to comply with each request to generate maximal muscle force,” participants “could not identify specific sensations of force, effort, or motion associated with the target muscles” (310). In an interview after the experiment, patient RB reported the following: “. . . there was no arm sense of effort or diaphragm sense of effort. . . all I felt was a generalized, ‘I'm trying to move,’ but nothing specific” (312). Patient DY reported that attempting maximum inspiratory effort “was mental effort.” A further report from DY: “I'm not sure the signal got there. I know my brain tried” (312).

These two studies sound the death knell for an application of the comparator account to experiences of trying. The experiences of trying reported in these experiences cannot be explained by matches between predicted and actual feedback, since there was no actual feedback. Nor can they be explained by internally simulated feedback, or anticipations of feedback. For such feedback, if centrally involved in the experiences of trying, should produce experiences of the feedback simulated or anticipated. That is, such experiences should be (presumably, sensory) experiences of things happening at the relevant bodily sites. But in the Lansing and Banzett study, very little sensory experience was reported. The experiences reported were largely directive in nature, concerning the direction of effort to the body parts. And in the Gandevia et al. experiment, experiences of trying were clearly distinct from (though seemingly causally linked with) the sensory experience of body parts moving. I take it, then, that there is good empirical reason to reject the comparator account for experiences of trying.^6^

These two studies are equally problematic for Wegner's view. Wegner maintains that the illusion of conscious will arises in part because actions are experienced as consistent with the agent's prior thought about the action (2002, chap. 3). The experiences of trying reported in self‐paralysis studies fail Wegner's consistency requirement. Like the comparator view, Wegner's is not well‐equipped to deal with the experiences reported in these self‐paralysis studies.

We have no good empirical reason to adopt a non‐constitutive view of the relation between experiences of trying and actual tryings. Should we be agnostics, or should we endorse a constitutive view? Attempts to answer this question must face up to the fact that we have nothing like a neurofunctional story precise enough to distinguish, at a neural level, between a number of loosely related agentive experience‐types: for example, experiences of an urge to A, experiences of preparing to A, and experiences of trying to A. The best we can do, at present, is to examine relatively rough‐grained correlations between agentive experiences and neural activities and to fit them into our best accounts of the neural activity that subserves action.

Regarding the rough‐grained correlations, the evidence reviewed above gives us somewhere to start. Experiences of trying to move appear to be causally upstream of, and to correlate reliably with, experiences of the body moving. Experiences of trying, then, are at least located in the right place to do what they seem to do – that is, to direct action.

In this connection it is important to note that in rejecting the comparator account I am not rejecting the importance of comparator‐type mechanisms in an account of motor control (see Wolpert and Kowato 1998). As I mentioned earlier in this section, I do not think experiences of trying need to involve experiences of fine‐grained motor control. Experiences of trying are experiences of directing effort towards the satisfaction of (often, relatively rough‐grained) intentions. Given this, the version of a constitutive view I find compelling does not need experiences of trying to be realized by the same neural activity that realizes motor control—that is, neural activity often described as motor commands, efference copies of motor commands, and on‐line predictions of upcoming sensory consequences based upon copies of motor commands. The neural activity in question might simply be the activity that realizes something like a conscious intention at work.

In connection with this point, consider two important brain‐stimulation studies. In the first, Fried et al. (1991) electrically stimulated the mesial precentral area (MPA) of the brains of thirteen surgical patients. The MPA contains the supplementary and the pre‐supplementary motor areas. Upon stimulation of the MPA the patients reported urges to move in various ways. As Desmurget and Sirigu (2012) observe, these urges had a few interesting properties. They were about specific (i.e., relatively fine‐grained) movements. They were not associated with active agency—often the patients simply reported that some part of their body was ‘about to move,’ not that they were about to move some part of their body. Finally, an increase in stimulation to the MPA produced actual movements of the body parts in question.

Contrast this with a study by Desmurget et al. (2009) in which experimenters electrically stimulated the inferior parietal lobule (IPL) of surgical patients. The IPL is upstream of the MPA, and is a part of the posterior parietal cortex (PPC). When experimenters stimulated the IPL, participants described experiences of desiring to move as well as experiencing “a will to move” (812). In contrast to the MPA‐stimulation experiences described above, IPL‐stimulation experiences were less specific (i.e., relatively rough‐grained), failing to include information about how the movement should occur. Further, these experiences were associated with active agency. Patients reported that they experienced a desire or a will to move the relevant body part, not that it was about to move. An increase in stimulation to IPL never led to actual movements—a result that Desmurget and Sirigu take to indicate that these experiences “occur upward of motor planning, at a time where the motor command has not yet been assembled” (2012, 1006). However, the increase in stimulation did lead to experiences as of the movement actually being performed, even though no movement occurred.

Given the increased stimulation, did the participants experience themselves as trying to make the movement they experienced? It would be good news for a constitutive view if they did, but the truth is that it is difficult to say from what Desmurget et al. report (although they interpret participant reports as conveying “the voluntary character of the movement intention and its attribution to an internal source, that is, located within the self” (812)). So I will not conclude that the Desmurget et al. study gives evidence that intense stimulation of the IPL induces experiences of trying. Even so, this study gives us evidence that an experience‐type closely related to an experience of trying can be induced by stimulation of the IPL.

These brain‐stimulation studies, in conjunction with the self‐paralysis studies discussed earlier, point in the direction of a constitutive view: the neural activity that realizes an experience of trying is just a part of the neural activity that directs real‐time action control. We have reason to believe, then, that experiences of trying play important causal roles in action control, as they seem to do.

This result—while, in my biased view, important and interesting—leaves unanswered a number of questions regarding normal experiences of acting. It is rarely the case, after all, that agents experience themselves trying without also perceptually experiencing effects of their trying. In what follows, then, I take up questions about the role of perceptual experiences in action control, and about the relation between perceptual experiences and the experience of trying.

## 3. Visual Experience and Zombie Action

Consider an action of reaching out to touch some visually located target, such as a cross. Brian O'Shaughnessy says this of such a case:

[O]ne keeps looking as one guides the finger, and does so right up until the moment the finger contacts the cross, and the reason, surely, is that sight is continually informing one as to where in one's visual field to move one's visible physical finger. (1992, 233)

At the outset of his seminal discussion of the role of conscious vision in action control, Andy Clark (2001, 496) quotes the same passage, and notes that by “sight” O'Shaugnessy is clearly referring to “conscious visual experience.” As such, O'Shaughnessy offers a good statement of the commonsense view on the role of conscious vision in action control. However, Clark goes on to argue that conscious visual experience plays no such role. According to Clark (and others, as we will see), though conscious visual experience might seem important for action control, it is “zombie systems”—systems that run outside the scope of conscious awareness — that do much of the work.

The conjunction of a constitutive view about experiences of trying and the view Clark and others endorse about conscious visual experience is, if not problematic, at least odd. If both views are correct, then (a) both experiences of trying and conscious visual experience seem to play important causal roles for action control, while (b) only experiences of trying in fact play the relevant roles. If both views are correct, then, conscious experience in action is in a sense disunified. Is this true? How—if at all—do agentive experiences such as the experience of trying relate to the perceptual experiences one has while acting?

In order to answer these questions, we need to get clearer on the nature of so‐called zombie action regarding vision. Zombie action arguments like Clark's are motivated and informed by Milner and Goodale's important work on the role of vision in overt (that is, bodily) action control. On their influential dual visual systems account, the human visual system is composed of two distinct information‐processing streams: the dorsal and the ventral stream. The dorsal stream is responsible for the on‐line control of overt action, and is not much involved with conscious visual experience. The ventral stream is responsible for conscious visual experience, and is not much involved with the on‐line control of overt action. The evidence for this account stems from two main sources, and is by now well‐known.^7^

First, work on patients with lesions to one of the two streams indicates a double dissociation between the operations of both. Patients with lesions to the ventral stream display visual agnosia: they can control overt actions related to objects in the environment even though they cannot recognize certain (presumably control‐relevant) features of these objects. Famously, for example, the patient DF can post a letter through a slot even though DF cannot recognize the orientation of the slot. Conversely, patients with lesions to the dorsal stream display optic ataxia. Although conscious vision remains unimpaired, optic ataxics are impaired in their ability to use vision to guide action related to the objects conscious vision picks out. For example, though a patient with optic ataxia will easily recognize a cup on a table, often her attempt to reach out and grasp the cup will be unusually awkward and laborious.

Second, research on the visual system of normal human adults indicates robust perception‐action dissociations that track functional differences between the ventral and dorsal streams. One basic finding is that illusions regarding the size of consciously perceived objects often have little influence on actions related to the objects. When presented with two identically sized circles, each surrounded by a ring of differently sized circles, participants reliably judge one circle larger than the other. Yet, when asked to grasp the circles, participants’ grip apertures reliably approach the actual size of the circles (Agliotti et al. 1995). This indicates that the ventral stream processes involved in the conscious visual illusion are in some way divorced from the dorsal stream processes involved in the control of the grasping action.

In an important series of articles (see Clark 2001; 2007; 2009), Andy Clark has argued that the dual visual systems account presents “a computationally challenging, empirically well‐supported, and philosophically important challenge to the view that conscious visual experience really does control daily, world‐engaging action” (2007, 566). Clark endorses the basic picture that unconscious encodings in the dorsal stream guide “fluent world‐engaging action” without assistance from encodings in the ventral stream, which support conscious visual states (2007, 566). Further, Clark argues that the dual systems account licenses the rejection of the following thesis (formulated by Wallhagen (2007)).

Experience Based Control‐general (EBC‐gen). Conscious visual experiences are typically utilized in the control and guidance of voluntary/intentional behaviors.

In Clark's view, conscious visual experience is useful primarily for the “reason‐and‐memory based selection of actions” (2007, 567). When it comes to overt intentional behavior, “even the gross heading and kinematics” are programmed “by the distinct representational structures proper to the dorsal stream” (573).

Like Clark, Wayne Wu (2008; 2013) supports the general dual‐systems picture. He argues that current evidence supports what he calls The Minimal Thesis:

The Minimal Thesis: Some visual representations that directly control and guide mundane bodily actions are unconscious. (2013, 218)

Wu denies that current evidence supports stronger versions of this thesis—e.g., a claim that *most* action‐controlling visual representations are unconscious. Even so, he comments that the truth of The Minimal Thesis “entails zombie action,” where (a) “any zombie action thesis implies that there are unconscious subject‐level visual states that control behavior,” (217–218) and (b) “a zombie action thesis affirms some form of epiphenomenalism regarding consciousness” (219).

In addition to Clark and Wu, Berit Brogaard has recently argued for the following two theses. First, “we cannot gain cognitive access to action‐guiding dorsal stream representations,” and second, “these representations do not themselves correlate with phenomenal consciousness” (2011, 1078). What do these theses indicate about the role of conscious visual states in control? Brogaard describes the implications as follows: while conscious vision can affect action, there is no conscious vision for action. This delicate distinction seems to depend on the claim that dorsal stream encodings are action‐guiding. The idea, presumably, is that dorsal stream representations are for action since they directly contribute to the on‐line control of action, while ventral stream representations merely affect action in the sense that their contributions—which are tied to functions such as object recognition—are mediated by the controlling operations of the dorsal stream.

To be clear, none of the above theorists (including Milner and Goodale) deny that ventral stream representations, and the conscious perceptual states closely associated with ventral stream representations, make a contribution to action. According to Milner and Goodale, we should distinguish between action planning and action programming. Action planning involves high‐level perceptual and cognitive processes directed towards selecting goals and identifying objects in the environment relevant to those goals. Action programming is typically said to encompass both the beginning of an action‐plan's implementation (i.e., the “pre‐specification of movement parameters” Milner and Goodale 2008, 776) and the ongoing real‐time guidance of an action's execution. For Milner and Goodale, ventral stream representations contribute to action planning (although the nature of the contribution has received far less attention than the contribution dorsal stream representations make to on‐line action control). It is the lack of conscious perception's involvement in action programming—sometimes glossed as action guidance or action control – that is supposed to be surprising and philosophically important.

At present, however, the exact nature of the philosophical importance remains unclear.^8^ In his 2001 paper, Clark motivated the thought that the dual visual systems account is philosophically important in two ways. First, Clark connected the dual visual systems account to ongoing debates over the existence of nonconceptual content in perceptual experience. Clark noted that many nonconceptualists justify their appeal to nonconceptual content by way of the assumption “that conscious visual experience provides the very information continuously used for visually based motor control” (496). This, of course, is an assumption the dual visual systems account challenges, and it gives us one way to understand the philosophical significance of the dual visual systems account. If correct, many arguments for nonconceptual content in perceptual experience do not succeed.

Second, Clark appealed to “a certain intuitive picture of the functional role of conscious visual experience” (499) that depends on an assumption Clark called the assumption of Experience Based Control:

Conscious visual experience presents the world to the subject in a richly textured way; a way that presents fine detail (detail that may, perhaps, exceed our conceptual or propositional grasp) and that is, in virtue of this richness, especially apt for, and typically utilized in, the control and guidance of fine‐tuned, real‐world activity. (496)

This assumption, and the intuitive picture it supports, involve appeals to how things *seem*. Clark wrote “although it may sometimes seem as if conscious seeing is what continuously and delicately guides our fine‐tuned motor activity, such online control may be largely and typically devolved to distinct, nonconscious, visual‐input‐using systems” (511). This is in line with Milner and Goodale, who claim that “The intrinsic interest of these demonstrations, in most cases, has been that they highlight surprising instances where what we think we ‘see’ is not what guides our actions” (1995, 77).

In what follows I focus on this second way of understanding the dual visual systems account's philosophical importance.^9^ According to this way, the dual visual systems account suggests that regarding the role of conscious vision in action, the way things seem is not how they are: commonsense is committed to the purportedly false view that what we (consciously) see guides our actions. I take it that this is why Wu claims that a zombie action thesis “affirms some form of epiphenomenalism about consciousness,” (2013, 219) and why Clark frames the philosophical importance of the relevant empirical work as undermining the view that visual experience “*really does* control . . . action” (2007, 566, emphasis mine). But what is the nature of this commonsensical commitment? And—assuming (for now: but see section four) the dual visual systems account is correct—how does commonsense go so wrong?

Consider a subject S who is disposed to falsely judge or form the belief that a visual experience plays an important causal role in overt action control at a time. If zombie action is true of us, we are in S's position. What explains S's epistemically faulty dispositions? One possibility is that these dispositions have nothing to do with the phenomenal character of S's visual experiences. S's epistemically faulty dispositions could be understood entirely in terms of the influence of background beliefs, belief‐formation processes, motivations to believe certain things about her own agency, etc.

In fact I do think that background beliefs (and perhaps motivation to see ourselves in a certain way) are a part of the explanation for the widespread surprise the dual visual systems account has elicited. Many seem to think that consciousness does much more than make *some* contribution to action control: consciousness is thought to do almost everything. On this view—which, like much folk psychology, is not likely to be precise—conscious processes are the primary controllers of whatever intentional movements I make. Now, if one has something approximating this view, there is precious little in conscious experience to contradict it. We rarely have any evidence that conscious visual experience is not involved in overt action control. In normal life we go about executing intentions, and since the execution of these intentions seems responsive to a primary feature of conscious experience, namely the conscious visual field, it is easy to infer that conscious vision must be important for overt action control. When we learn that, for example, intentional actions of grasping sometimes involve nonconscious adjustments of grip size, this sits uneasily with a background belief that conscious processes are the primary controllers.

I think the above is part of an explanation for the surprise that many express regarding the dual visual systems account. But I doubt it is the full story. To see why, consider the interesting possibility that S's epistemically faulty dispositions are to be understood in part by appeal to the phenomenal character of her visual experiences (as opposed to the absence of any defeaters in that phenomenal character). On this possibility, S's visual experiences are in some way misleading.

With this possibility in mind, consider the following template for zombie action.

Zombie Action. There is a class of conscious experiences that (1) partly in virtue of their phenomenal character, dispose their subjects to judge (or form the belief) that tokens of the class play an important causal role in overt action control at a time, and (2) do not play the causal role subjects are disposed to judge or believe they do.

As elucidated, Zombie Action is quite general: an interesting zombie action thesis will involve specification at three places. First, we want to know more about the type of conscious experience at issue. For example, much of the discussion surrounding the dual visual systems account involves an apparently intramodal thesis, concerning conscious vision alone. But of course one might consider zombie action theses regarding other modalities, or regarding various combinations of modality. Whether these are interesting will depend in part on the type(s) of conscious experience at issue.

Second, we want to know more about the scope of the class of conscious experiences at issue. A zombie action thesis regarding a very restricted class of conscious experiences might not be very interesting. In this connection, notice that while early discussions of the dual visual systems view sometimes made the relevant class (of conscious visual experiences) look rather large, in recent years one finds the scope of the relevant class diminishing. Clark (2007) denies that conscious visual experiences are *typically* utilized to control action. Wu (2013) claims only that *some* controlling visual representations are unconscious. Whether such claims are philosophically important depends, in part, on the scope of the class.

The third place at which specification is required concerns the phenomenal character of the relevant conscious experiences. Much of the extant literature says very little about this: here, it seems, Zombie Action has a genuine contribution to make. In what follows, I consider three options for a zombie action thesis about vision.

Consider, first, a visual experience with an illusory phenomenal character as of playing an important causal role in overt action control at a time. If such experiences exist, it is not difficult to imagine that they would generate dispositions to judge that visual experiences play important causal roles in overt action control. The problem with such experiences is that, at least as applied to visual experience, it is difficult to understand the nature of the phenomenal character at issue. What is it to have a visual phenomenal character as of playing an important causal role in overt action control at a time? One possibility is that this description attributes self‐referential causal content to a conscious visual experience. On this possibility, a conscious visual experience represents *itself* as causing certain things. (Notice, in this connection, that a conscious visual experience that simply represented *the agent* as causing certain things carries no commitment to its own role in overt action control.) This is possible, but in my view implausible. Although some have argued that visual experiences sometimes possess causal content (see Siegel 2005), I know of no one who defends the view that visual experiences sometimes possess self‐referential causal content.^10^

A second possibility, discussed briefly in section two, is Susanna Siegel's idea that some visual experiences are experienced as *mandates*. Recall that for Siegel, experienced mandates are experienced affordances which include “a high degree of felt solicitation” (2014, 55) to perform some relevant action:

[S]uppose in the midst of an important conversation the tuft of hair keeps falling over your interlocutor's eye, obstructing proper communication by interfering with eye contact. You might well experience the hair as an obstacle that should be moved away to allow for fuller eye contact. (54)

As Siegel recognizes, Hubert Dreyfus appeals to something like experienced mandates in his descriptions of the phenomenology of skilled action. Consider the following passage (quoted by Siegel as well) in which Dreyfus is describing skilled tennis:

[W]hat one experiences is more like one's arm going up and its being drawn to the appropriate position, the racket forming the optimal angle with the court—an angle one need not even be aware of—all this so as to complete the gestalt made up of the court, one's running opponent, and the oncoming ball. One feels that one's comportment was caused by the perceived conditions in such a way as to reduce a sense of deviation from some satisfactory gestalt. (2002, 379)

In my view this kind of phenomenological description is at least roughly correct, and the fact that our experience of acting is sometimes like this contributes to the general surprise generated by the dual visual systems account. Experienced mandates have a kind of motivational force, and as such certainly seem to play the kind of causal role felt motivation often does in action control. When one experiences a visual mandate while acting, it is as though one's conscious perception of the visual scene, and with it consciously felt solicitations to move in certain ways, are themselves responsible for the fact that one moves in those ways. It would be surprising if this class of visual experiences had a phenomenal character as of soliciting one's actions, and yet—as a zombie action thesis maintains—these experiences played no causal role in the on‐line production and direction of these actions.

It must be noted, however, that it is unclear whether visual experiences qua visual could have the kind of motivational force Siegel ascribes to experienced mandates (Siegel herself remains neutral here). Ascribing motivational force to a visual experience is likely to strike many as unnecessary: arguably, one might visually experience an affordance – one might experience some hair as to‐be‐moved (or as moveable)—without experiencing any motivation to do so. Why not say that an experienced mandate is a combination of experiences: perhaps a visually experienced affordance and a desire or urge to perform an action suggested by the affordance? If this is the right way to describe an experienced mandate, then a strictly visual version of Zombie Action will not apply. We will want to articulate a multimodal thesis, combining visual and motivational experience. I return to this issue in section five.

The above discussion of action‐affordances suggests a third possibility. According to Bence Nanay visual experience often contains *action*‐*properties*: properties that “can't be fully characterized without reference to one's action” (2011, 311). These are distinct from affordances, which many claim are perceived directly.^11^ For Nanay, action‐properties are attributed to objects, form a normal part of our perceptual experience of many (but not all) objects, and enable action related to those objects (see Nanay 2013, chap. 2). Examples include experiencing a tree as climbable, experiencing a newspaper as apt for fly‐killing, or experiencing a slice of cake as edible. These experiences have no motivational component, but they are closely connected to action. Suppose what is controversial: that Nanay is right and some visual experiences contain action‐properties. These experiences would have a phenomenal character apt to produce judgments or beliefs about their role in action control. For example, a ball is flying towards you and you intend to hit it with your head (towards the goal). Say that you experience the ball as headable. You see the ball take an odd swerve, which changes the action‐property: the ball is no longer headable, but it is shoulderable. That is, you can still direct the ball towards the goal by hitting it with your shoulder. So this is what you do. Sensibly (it seems), you form the belief that your visual experience of the ball—the experience that the ball post‐swerve became shoulderable—played an important causal role in your shouldering the ball. If the visual experience did not play this role, we have a case of visual zombie action.^12^ Conscious visual experience will be misleading in the sense that it licenses the formation of false judgments or beliefs about its role in action control.

Whether any zombie action thesis is true, of course, depends on claim (2) – the claim that the relevant experiences do not play the causal role subjects are disposed to judge or believe they do. I have not yet evaluated any evidence relevant to the dual visual systems account. In the next section I do so.

## 4. Does Conscious Visual Experience Contribute to Action Control?

Arguments for visual zombie action assert that commonsense is committed to the false view that what we consciously see guides our actions. Recall that this claim about guidance is ultimately a claim about action programming: no one denies that conscious vision has a role to play in action planning.^13^ Does conscious visual experience play no role in action programming?

Recent criticisms of the dual‐visual systems account indicate that conscious visual experience does have some role to play in action programming (Mole 2009; Schenk et al. 2011; Briscoe and Schwenkler forthcoming). For example, it seems that certain sophisticated behaviors require a good deal of interaction between dorsal and ventral streams (Schenk and McIntosh 2010). Further, in recent work Wayne Wu (2014) and Berit Brogaard (2012) offer plausible arguments that information in the dorsal stream contributes to the content of conscious visual experience. And in a forthcoming paper—one which assesses the evidence in much more depth than I can here—Robert Briscoe and John Schwenkler make a compelling case that “there is no general, empirically‐based objection to the notion that consciously encoded information is used to control bodily actions” (forthcoming).

Consider a recent study by Gonzalez et al. (2008). In this study researchers had participants grasp objects presented against the backdrop of a Ponzo (e.g., size‐contrast) illusion with either a practiced, skilled grasping motion (using index finger and thumb) or an unpracticed, awkward grasping motion (using ring finger and thumb). While the practiced motion was not subject to the size illusion, the awkward motion was, suggesting that awkward, unpracticed or novel movements rely on (potentially conscious) ventral stream representations.

In another relevant study, McIntosh and Lashley (2008) had participants reach for and grasp matchboxes across trials. On later trials the matchbox differed in size from earlier trials, even though the cover of the matchbox remained similar. They found that the expected size of the matchbox – judged, presumably, thanks to object recognition processes supported by the ventral stream – influenced both the shaping of the hand and the amplitude of the reach. As McIntosh and Lashley note (2444), this suggests that ventral stream encodings are utilized to program actions related to specific kinds of objects.

Finally, consider a study by Caljouw et al. (2011). In this study participants hit a ball towards the vertex of a Müller‐Lyer illusion. In one condition, the illusion did not change throughout the action of hitting the ball. In other conditions, experimenters flipped the tails of the Müller‐Lyer arrows such that the illusion—and with it the place at which participants were consciously aiming – changed. They did this after the hitting motion had begun. Experimenters found that changing the consciously perceived illusion significantly impacted participant hitting actions. More specifically, mean impact velocity of the hitting device changed in the direction of the illusory change of the target. It looks like participants behaved like this: aim at a certain location; begin swinging; consciously see the target change locations; adjust hitting velocity to compensate. Caljouw et al. report: “any abrupt environmental change in the target area invoked online visual control that is affected by the illusion” (1139).

One might complain that ventral stream involvement in behavior does not entail the involvement of conscious vision: not everything in the ventral stream informs conscious vision. This is true, but recall that the case for the dual visual systems account depends on a strong ventral/dorsal distinction. The best reason to believe conscious vision plays no role in action programming is that conscious vision is located in a part of the brain that plays no role in action programming. The above studies indicate that in at least some circumstances, we have good reason to doubt that this reason obtains. Regarding the Caljouw et al. study in particular, it seems difficult to deny that conscious vision is playing some role. Maintaining that the ventral stream information utilized by participants in this study is non‐conscious information, in spite of the fact that the information is consistent with the consciously perceived illusion, is ad hoc.

So it looks like conscious visual experience sometimes plays important roles for action control. Are the roles it plays consistent with commonsense beliefs about the role of conscious vision? Perhaps not. In section three I noted that many seem to think that consciousness does much more than make *some* contribution to action control: consciousness is thought to do almost everything. Add to this the observation that there is little in conscious experience to contradict this view, and it is easy to maintain that commonsense is guilty of overgeneralization from cases in which conscious vision plays some role to cases in which it does not. Accordingly, the evidence that undergirds the dual visual systems account might legitimately reveal something surprising about human agency. Conscious visual experience is less important than we thought it was.

Is this discovery at odds with the phenomenology of acting? That is to say: is conscious visual experience misleading? Recall a point I made about the experience of trying. This experience is relatively rough‐grained, its neural realizers fairly high up within the action control hierarchy. Arguably, the same is true of visual experience in action. I doubt that many people have a phenomenology as of visual experience informing micro‐adjustments to, e.g., grip aperture (perhaps by attributing action‐properties). If this is right, conscious visual experience is not misleading, even though the results driving the dual visual systems account are genuinely surprising.

## 5. The Multimodal Experience of Acting

I began this paper by describing an activity—looking at the hand, lifting it, slowly wiggling each finger—that involves experiences in at least three modalities. There is visual experience of the hand moving, there is broadly haptic (or proprioceptive) experience of the hand moving,^14^ and there is an experience of trying to move the hand. I have argued that at least two of these experience‐types do what they (phenomenally) seem to do (there is not space to fully treat haptic perceptual experience here). They make causal contributions to at least some actions. The question I wish to ask in this section concerns how these experience‐types relate to each other in full‐blown experiences of acting. The answer I wish to defend can be stated as follows.

Experience of acting. The experience of acting typically consists of temporally extended experiences from more than one modality. These experiences are easily associated with the action being performed in virtue of the fact that their contents fit coherently into the agent's broader plan for action. And their contents fit coherently in virtue of the fact that they are functionally integrated and structured by what the agent is trying to do.

In defending this view of the experience of acting, I will leave a number of issues on the table that a longer discussion might address. First, I say nothing about whether any particular experience‐type (e.g., the experience of trying) is necessary for an experience of acting. Second, I say nothing about whether any particular experience‐type (e.g., a perceptual experience of success) is sufficient for an experience of acting. Third, I say nothing about how this account relates to an account of experiences of error while acting (though in my view an extension to such cases should not be difficult).

In addition, I remain neutral regarding the nature of the multimodal experiences that constitute a typical experience of acting. On this issue, we have at least three options. First, perhaps the experiences that constitute an experience of acting do so in only a loose, associative sense. Consider Matthew Fulkerson's view on multisensory experiences. According to Fulkerson, “Multisensory perceptual experiences do not involve the direct predication of features associated onto individual perceptual objects . . . What we experience is a higher‐order association between sensory experiences” (2011, 506). Everything I say below is consistent with this view.

A stronger view—one that, for the record, I find very plausible—is that the experience of acting involves what Casey O'Callaghan calls intermodal feature binding awareness. In intermodal feature binding awareness, “features consciously perceived through different modalities can perceptually appear to be bound and thus to belong to the same thing” (2014, 76). The result is that some conscious experiences “may not be factorable without remainder into co‐conscious modality‐specific components that could have occurred independently from each other” (88). Perhaps, then, agentive, visual and haptic experiences are bound to the body‐in‐motion in the way that visual and auditory experiences appear to be bound to an extended event of someone uttering a sentence.

A third, even stronger possibility, is that some multimodal experiences have an amodal component. Perhaps, for example, agentive, visual and haptic experiences are recoded into an amodal format and bound to an extended amodal experience of acting.

What I say below is consistent with all these possibilities, but commits to none of them. What I am committed to is the view that experiences of acting are typically constituted by experiences from multiple modalities (including whatever cognitive or agentive modality is responsible for the experience of trying), that these experiences are functionally integrated, and that their contents are thus easily and coherently associated with the agent's activity over a relevant period of time. In a sense, this view is general enough to be somewhat unsurprising. Even so, I have not seen such a view on the experience of acting articulated in the way I wish to do so.

It turns out that there is a good deal of evidence in favor of a multimodal account of the experience of acting. My discussion is therefore selective. I focus on empirical work that helps fill in the general picture I endorse.^15^

The first kind of relevant evidence is evidence for functionally integrated processing between sensory modalities. For example, much bodily experience depends in various ways on integrated processing of visual and haptic information.^16^ This is well llustrated by the Rubber Hand Illusion. This illusion is induced by (a) occluding a subject's hand from view while stroking it with a tool, and (b) having the subject watch as a rubber hand, placed some distance away from the subject, is simultaneously stroked. The result is typically that the subject feels that the hand she sees is her hand, strangely located *over there*. The Rubber Hand Illusion thus seems to result from an interaction between visual and haptic information in which visual signals dominate haptic signals to determine the hand's location.

A second body of relevant evidence indicates that visual object recognition is influenced by affective or motivational information regarding an object's valence – i.e., whether the object is or might be attractive, repellent, helpful, harmful, and so on. Indeed, such motivational information is closely integrated with (even very low‐level) information processing in visual cortex, such that in practice disentangling visual from motivational experience is quite difficult. In a fascinating review of the evidence, Barrett and Bar (2009) argue that “affective responses signalling an object's salience, relevance or value do not occur as a separate step after the object is identified—affective response assists in seeing an object as what it is from the very moment that visual stimulation begins” (1326–1327).^17^ Viewing the evidence, it is difficult to disagree with Barrett and Bar that (a) affective processing, largely subserved by orbitofrontal cortex, is functionally critical for the visual processing that takes place in visual cortex, as well as (b) much perceptual experience in healthy adults—especially experience of objects—includes a motivational component. If Barrett and Bar are right, then much normal perceptual experience—experience of faces and objects and scenes—is richly multimodal in at least the weak, associative sense described above (indeed, Barrett and Bar often refer to the representations that underlie the conscious perception of objects as “multimodal representations”).

This kind of evidence indicates that integrated multimodal processing undergirds much conscious perception. A multimodal account of the experience of acting requires more than this. What we need is further evidence indicating that while acting what an agent is doing (i.e., what she is trying or intending to do) structures her perceptual experience. Fortunately, there is ample evidence that actual trying—the actual execution of an intention—structures an agent's utilization of perceptual feedback in a coherent way.

Return to the integration of visual and haptic information for hand location. Although vision often dominates haptic perception, the integration of visual and haptic information is sensitive to whether or not the movements of the hand are self‐generated. According to the optimal integration model developed by Robert van Beers and colleagues (van Beers et al., 1999; van Beers et al., 2002), though visual information is often weighted more heavily than haptic information, haptic information receives relatively more weight when movement is self‐generated. This is evidence that trying to move the hand structures the way that perceptual information relevant to the action is processed.

Further evidence comes from an experiment utilizing binocular rivalry. In binocular rivalry, each eye sees a different stimulus, and as a result conscious vision oscillates between stimuli. Maruya et al. (2007) showed participants two stimuli. One eye saw a sphere of rotating dots. The other eye saw a flickering sphere of black and white grating. In the experiment, participants were able to drag a mouse across the rotating dots, thereby controlling that sphere. Interestingly, doing so had two effects. When the rotating sphere was in conscious vision, controlling its rotation caused the sphere to remain in consciousness for longer than normal. And when the rotating sphere was not in conscious vision, controlling its rotation caused the sphere to return to conscious vision more quickly than normal. In these cases, what the agent is trying to do has a structuring influence on conscious perception.

The same thing seems to be true regarding temporal experience in action. In the well‐known intentional binding effect (Haggard et al. 2002), what an agent is trying to do structures the temporality of the perceived consequences of the trying. More specifically, in conditions of voluntary action (as compared to non‐voluntary conditions) the perception of an action's onset and a perceived consequence of action are compressed in consciously experienced time.

Finally, it is worth mentioning work on sensory attenuation. Sensory attenuation refers to lower levels of felt or, in some cases, cortically measured intensity for a given sensory stimulus. One general finding is that when action is self‐generated, relevant sensory stimuli are attenuated (Helmchen et al. 2006; Martikainen et al. 2005). It is thought that this attenuation reflects the fact that when movement is self‐generated, the related sensory stimuli are expected, so the agent (or various sub‐personal mechanisms responsible for attenuation) can afford to shift attention elsewhere. For a particularly clear demonstration of this, consider a recent study by Timm et al. (2013). Timm and colleagues had participants press a button which produced an expected auditory effect. In one condition, participants voluntarily pressed the button. In a second condition, experimenters applied transcranial magnetic stimulation to the motor cortex which caused an involuntary button press. The voluntary button press led to auditory attenuation; the involuntary button press did not.

A wide range of evidence thus supports a multimodal account of the experience of acting. In paradigmatic cases, the experience of acting is (at the very least) a temporally extended, co‐conscious collection of agentive and perceptual experiences, functionally integrated and structured both by multimodal perceptual processing as well as by what an agent is, at the time, trying to do.

## 6. Conclusion

I began with an assertion. Consciousness appears, in some very vague way, to play an important causal role in action control. I have examined this vague appearance from a few angles, hoping to get clearer regarding what it might indicate. I have argued that a unique kind of agentive experience, the experience of trying, does what it seems to do. An experience of trying directs effort towards the satisfaction of an intention because the neural realizers of such an experience are not distinct from those that realize the actual direction of effort towards the satisfaction of an intention.

Further, I have assessed recent empirical work on the role of conscious visual experience for action control. There are a few ways this work might undermine commonsense. I have attempted to get clearer on the possibilities by exploring how the phenomenal character of visual experience might give rise to false judgments or beliefs about how visual experience contributes to action control. I conceded that the relevant empirical work undermines commonsense. It does so, however, not because visual experience is misleading, but because we assume conscious visual experience does more for action control than it actually does. This is not to say that visual experience does nothing. I have also argued that in at least some circumstances, conscious visual experience makes important contributions to action control.

Finally, I have articulated a multimodal account of the experience of acting that begins to explain how experiences of trying relate to perceptual experiences in action. In paradigmatic cases, the experience of acting is (at the very least) a temporally extended, co‐conscious collection of agentive and perceptual experiences, functionally integrated and structured both by multimodal perceptual processing as well as by what an agent is, at the time, trying to do.^18^

## Notes

1 The experience of trying is similar, I claim, whether the action is bodily or mental. For an argument that control over mental action works via similar processes as control over bodily action, see Shepherd (2014).

2 Multiple conversations with philosophers about these issues reveals that while many agree with me that the experience of trying is manifest, some profess puzzlement.

3 This is consistent with a view of tryings according to which tryings are identical with the effects of a proximal intention's normal functioning—i.e., its initiating, guiding and sustaining of action (see Adams and Mele 1992). It is perhaps worth mentioning that trying is necessarily intentional: it is what successful and failed intentional actions share. When one successfully A‐s, it is in part because one has tried to A. And when one has tried to A and failed, one has still done something intentionally – one has tried. Thus understood, trying is at the foundation of agency. Since someone is an agent only if they have the capacity to act, someone is an agent only if they have the capacity to try.

4 Some tryings might include a non‐neural (e.g., a bodily) component. I omit this qualification here and in what follows.

5 A tourniquet applied to the forearm kept the vecuronium from paralyzing one hand. This allowed participants to communicate with researchers via finger movements on this hand alone.

6 Thanks to an anonymous referee for pressing me on this point. The same referee raises the issue of an agentive experience‐type's direction of fit. Although many agree that (at least many) agentive experience‐types have intentional content, there is disagreement over how to understand their intentional structure (see Bayne 2011). Some hold that like intentions and desires, agentive experience‐types have a directive or world‐to‐mind direction of fit. Others hold that like beliefs, agentive experience‐types have a descriptive or mind‐to‐world direction of fit. Finally, some have suggested that at least some agentive experience‐types could have both directions of fit. In my view the most natural understanding of experiences of trying attributes at least some directive intentional structure to them. Indeed, if the neural realizers of experiences of trying are not distinct from the neural realizers of actual tryings, this is what one would expect. Even so, my main aim here is to establish the constitutive view: I take it further work is required to establish what kind of intentional structure experiences of trying possess.

7 For book‐length treatments of the relevant evidence, see Milner and Goodale (1995; 2004). For recent positive discussions of the dual‐visual systems view, see Milner and Goodale (2008) and Clark (2009). For recent critical discussion, see Schenk and McIntosh (2010) and Briscoe and Schwenkler (forthcoming).

8 For some discussion of this point, see Wallhagen 2007, Grünbaum 2012.

9 This is not to say that the connection between nonconceptual content and the dual visual systems account is uninteresting or unimportant. But it is worth noting two things here. First, Clark formulated the assumption of Experience Based Control so that it was neutral on the issue of nonconceptual content. And in subsequent work on the dual visual systems account this issue has received less attention. This suggests (to me) that for many, the challenge to commonsense is of paramount importance. Second, though one need not invoke how things seem (or, as a referee notes, anything about an experience's phenomenal character) in order to connect action control and nonconceptual content, at least some of the attempts to make this connection appear, in my view, to invoke either how things seem or phenomenal character. Clark quotes Jose Luis Bermudez, who writes “Theorists have been attracted to nonconceptual content by the thought that the richness and grain of perceptual experience is not constrained by the concepts that a believer might or might not possess” (1998, 50). In my view, the language of richness and grain is at least in part about what perceptual experience seems to be like—about its phenomenal character. If beliefs about phenomenal character do (sometimes) motivate claims about nonconceptual content, the Zombie Action thesis developed in this paper will be relevant (both to the beliefs and to the claims). Regarding this issue, and those in the main text surrounding it, I owe thanks to an anonymous referee for challenging and helpful comments.

10 John Searle (1983) does argue that some mental states (intentions) have self‐referential causal content. But the states he has in mind have a world‐to‐mind direction of fit. The visual states in question above would seem, instead, to have a mind‐to‐world direction of fit.

11 Nanay notes that proponents of affordances “deny that we perceive objects and attribute properties to them,” and affirm that “what we perceive are affordances, not objects” (2013, 11). By contrast, Nanay claims “that among the numerous properties we attribute to the perceived objects, some are action‐properties: properties the representation of which is necessary for the performance of one's action” (11–12).

12 An anonymous referee suggests that this example is consistent with Milner and Goodale's view that conscious perception is important for action planning but not action programming. Perhaps this is right. However, little is said in this literature regarding the specific role of action planning for action control once action implementation is underway. If anything there is a tendency for theorists to assume that once action is underway whatever changes to an action must be made will be made via dorsal stream processes (see, e.g., Milner and Goodale 2008, 776). In this connection, it is worth noting that one strand of evidence taken to favor the dual visual systems hypothesis concerns real‐time action changes: it is often noted that agents will non‐consciously change the direction of their reach to respond to changes in target location. What we do not often see are examples of real‐time action changes accompanied by visual experiences relevant to the change. I discuss one such case in section four.

13 I take this distinction on board here for the sake of argument. But in fact I think that a more complete understanding of action control undermines the force of this distinction. Action planning processes do not cease functioning at the moment of action‐initiation: action planning processes will often be crucial to the successful execution of many intentions. So it is misleading to treat action programming processes as important for action guidance to the exclusion of action planning processes (for more on this point, see Shepherd forthcoming).

14 It is a matter of some controversy how to best taxonomize haptic perception: as I will understand it here, haptic perception involves proprioception (roughly, the sense of body position), kinesthetics (roughly, the sense of body movement), and touch (roughly, a kind of contact sense). More precision regarding haptic perception would require discussion of difficult issues regarding sensory individuation, as well as the nature of touch (Ratcliffe 2012) and the nature of kinesthesia and proprioception (Fridland 2011). I will not do that here.

15 Some of what follows applies or extends similar arguments offered in Shepherd (forthcoming).

16 Indeed, Frederique de Vignemont has recently argued that bodily experience is constitutively multimodal, in the sense that “multisensory binding is a constitutive component of the etiology of bodily experience” (forthcoming).

17 Thanks to an anonymous referee for pointing me to this evidence.

18 Thanks to three anonymous referees at *Noûs* for excellent and challenging comments. Thanks to one anonymous referee at *Australasian Journal of Philosophy* for truly excellent feedback on an earlier version of this paper. Many audiences saw presentations of ideas that ended up in this paper, and thanks are due to all of them: I have in mind audiences at the 2012 Tuscon consciousness conference, CUNY‐Graduate Center's Cognitive Science Group, The University of Edinburgh, the 2013 APA‐Pacific, the 2013 Society for Philosophy and Psychology meeting, Oxford's Philosophy of Mind work‐in‐progress group, and Università degli Studi di Milano. For convsersations, comments, and inspiration, sincere thanks as well to Tim Bayne, Jake Berger, Andy Clark, Anil Gomes, Marcela Herdova, Al Mele, Alex Moran, Myrto Mylopoulos, David Papineau, Matt Parrott, Ian Phillips, Oliver Rashbrook‐Cooper, David Rosenthal, Susanna Siegel, and Wayne Wu.
